# P-314. Perspectives of Women who have Migrated to Paris, France from Sub-Saharan Africa on Awareness, Promotion, and Implementation of HIV Pre-Exposure Prophylaxis

**DOI:** 10.1093/ofid/ofaf695.533

**Published:** 2026-01-11

**Authors:** Kaylie Miller, Julie Castaneda, Geoffroy Liegeon, Samantha A Devlin, Jessica Ridgway, Manda Victoria, Andres Ramirez Zamudio, Amy K Johnson

**Affiliations:** University of Chicago, Chicago, IL; Lariboisière Hospital, Paris, Ile-de-France, France; Hôpital Saint-Louis, Paris, Ile-de-France, France; University of Chicago, Chicago, IL; University of Chicago Medicine, Chicago, Illinois; Saint-Louis Hospital, Paris 10e Arrondissement, Ile-de-France, France; Saint Louis Hospital, Paris, Ile-de-France, France; Ann & Robert H Lurie Children’s hospital of Chicago, Chicago, Illinois

## Abstract

**Background:**

Women who have migrated from sub-Saharan Africa (WMSSA) represent 75% of new HIV diagnoses among women in France, with a projected 30-50% of those women acquiring HIV in France. Despite HIV pre-exposure prophylaxis (PrEP) being available and the cost subsidized, WMSSA represent only 5% of all PrEP users in France in 2024. We aimed to address this gap by better understanding knowledge and perspectives of HIV prevention modalities and how focused messaging may increase PrEP uptake among WMSSA.

**Methods:**

We conducted an exploratory qualitative study with focus groups and a short demographic survey among WMSSA. Focus groups occurred between October 2024 and January 2025. Women without HIV were recruited from the Lariboisière family planning center in Paris, France. Data analysis involved thematic coding and was informed by the social ecological model and the theoretical framework of acceptability.

**Results:**

Three focus groups were conducted (N=11). The median age of participants was 34. Most women (8/11, 73%) had migrated from Nigeria. Participants had been living in France for a median of 9 years. On the individual level, women expressed beliefs and experiences of HIV stigma (e.g., not wanting to be in close proximity to a person with HIV, sharing utensils as a cause of HIV, being able to visually discern HIV status, etc.). Most women were unaware of PrEP, but responded positively once it was explained. There was an emphasis on safety as a driving factor behind PrEP interest. Regarding implementation strategies, participants felt outreach would be most effective through social media (i.e., TikTok and YouTube) and brochures at health centers. However, participants felt that they needed a baseline understanding of PrEP to interpret advertisements and favored advertising that focused on people of all genders and races. In terms of modality acceptance, participants viewed long-acting injectable PrEP and the vaginal ring as favorable; there were many concerns about adhering to a daily pill.

**Conclusion:**

WMSSA in our study were generally unaware of PrEP but highly interested in long-acting methods to increase safety from HIV acquisition. More studies are needed to evaluate the translation from outreach and focused messaging to uptake of PrEP among WMSSA.

**Disclosures:**

Manda Victoria, MD, GILEAD: Advisor/Consultant Andres Ramirez Zamudio, N/A, MD, MPH, Viiv: Advisor/Consultant

